# The Importance of Clinical Study of the Lower Animals

**Published:** 1912-10

**Authors:** Charles Haase


### The Importance of Clinical Study of the Lower Animals.

Our attention has been called anew to this subject by a remark of Dr. Aristides Agramonte, that yellow fever was solely a human disease and that it concerned merely man and the stegomyia. Certain observations as to the date of infectivity of this musquito after drawing blood from a yellow fever patient, render it probable that the former must actually contract the disease before spreading it. While it is ludicrous to think of the stegomyia as a patient attended by physicians carefully noting symptoms, a little reflection will convince us that if we really could determine the clinical progress of yellow fever in the musquito, or if, on the other hand, we could definitely declare it to be in normal health after drawing infected blood, we should undoubtedly be much nearer the discovery of the ultimate germ and we should also be much better prepared to prevent the development of human cases.

Veterinary medicine does deal with the clinical side of disease in a number of lower animals, valuable commercially or invoking the aid of their masters as domestic pets. But the clinical study of disease in the lower animals might be of much greater service to science and to humanity if carried on systematically without regard to size, number of legs and presence of a backbone. Certain forms of clinical study have proved of value in the detection of tuberculosis in bovines, tetanus in horses, rabies in dogs, etc.; trichinosis in swine. Bovine variola—vaccinia—is a disease of transcendent importance. It is not impossible that a thorough study of grease and of ovine variola might develop further aids to prophylaxis and even therapeutics of human variola. The detection of trichinæ by routine post mortem microscopic examination—the details being mainly in the hands of comparatively untrained assistants—does not impress us as very reliable. It is possible that purely clinical observation of swine, ante mortem, might prove more reliable. There is unquestionably more or less danger of tuberculous infection from cats and dogs; considerable danger from monkeys and guinea pigs; possibly some from pet birds. In the case of mammals, it would seem that barring the hair which can easily be shaved off, pulmonary tuberculosis could be diagnosed at least as easily as in human infants. The spread of diphtheria from cats is established, yet actual clinical diagnosis is almost never employed. Recently, attention has been called to the possibility of anterior polio-myelitis in domestic animals and we have personally heard a report, from lay sources, it is true, of fairly typic infantile paralysis in a kitten. Some years ago, long before the treponema was known and when syphilis was regarded as a purely human infection, we attended a man with tertiary syphilis. His pet cat used to eat meat which the master had chewed and spit out. The cat had the typic raucous voice, falling of hair and glandular swellings of human syphilitics and was said to have passed through the ulcerative stage. At the time, we considered it a joke to say that the cat was syphilitic. In the light of recent discoveries, we are by no means certain that the condition should not have been taken seriously.

None of the modern vaccinations are closely analogous to antivariolar vaccionation either in probable detailed explanation nor in protective power. In other words, there is no other instance known of a corresponding naturally mitigated animal disease which will protect against the human infection. All of the other exanthemata appear to be closely analogous to small pox. If somewhere in the animal kingdom not necessarily among mammals nor even vertebrates, a vaccination against scarlatina could be found, no one would laugh at a clinical study of disease in lower and even minute animals.

---

With the rapid extension of lodge practice, contract practice, Health Board, hospital and dispensary abuses it is becoming more and more difficult for physicians to make a living. It is time that we look the situation square in the face and consider what is best for the profession instead of holding up our hands in horror and shouting, "It is not ethical," when one mentions the business side of medicine.

Those who hold up their hands in horror are those who are well established or have made their "pile" and they do not need to think about the financial side of the practice of medicine. They are selfish and do not concern themselves about the welfare of the profession.

CHARLES HAASE.

